# The Syrup of Iron Chloride

**Published:** 1892-03

**Authors:** G. W. Weld

**Affiliations:** New York


### International Dental Journal.

Vol. XIII. March, 1892. No. 3.

### Original Communications.[1]

### THE SYRUP OF IRON CHLORIDE.[2]

BY G. W. WELD, M.D., D.D.S., NEW YORK.

Mr. President and Gentlemen of the Odontological Society of Pennsylvania,—I wish to gratefully express my convictions that no small share of any success which I may have met with in my experiments is attributable to the circumstance of my having had the assistance of one or two distinguished chemists, and the friendly encouragement of a goodly number of physicians residing in New York. Having been led by special circumstances, some seven years ago, to begin a series of experiments, I found it impossible to continue without sacrificing a large portion of time usually allotted to repose, at no inconsiderable cost of both health and spirits. The experiments have been associated with certain other disadvantages, which accounts for the imperfection of some of the first productions.

From the testimony of several physicians who have lately employed the preparation in their practice, I am led to believe that I have finally succeeded in changing an old reliable compound of iron into what may be considered a new compound, concerning which the merits are worthy the thoughtful consideration of the medical and dental professions.

---

[1] The editor and publishers are not responsible for the views of authors of papers published in this department, nor for any claim to novelty, or otherwise, that may be made by them. No papers will be received for this department that have appeared in any other journal published in the country.

[2] Read before the Odontological Society of Pennsylvania, January 9, 1892.

Whatever may be thought of the SYRUP OF IRON CHLORIDE, I wish to observe just here that the experiments have been conducted in a grave and earnest spirit, my only aim having been to produce a compound of the chloride which could be presented in a potent and permanent form (not emasculated), easily assimilated, and accompanied with all the virility of the tincture without its objectionable features.

For a proper understanding of the subject (as the syrup of iron chloride is nothing more nor less than the tincture of the chloride of iron slightly modified), permit me to introduce the same by first making a few remarks on the tincture.

The tincture of the chloride of iron is made from the liquor ferri chloridi, and contains thirty-seven and eight-tenths per cent. of the dry chloride. In making the tincture of the chloride, thirty-five parts of the liquor are added to sixty-five parts of alcohol. Attfield says "that the liquor which is used in making the tincture contains much free acid," which, it is claimed by some authorities, is necessary to prevent the precipitation of the basic salts of iron, and this free acid contained in the tincture constitutes one of the objections which has been raised against its use, because of its destructive energy upon the enamel of the teeth.

Before an audience composed chiefly of dentists and physicians it would seem to be almost the triteness of an observation to comment on the pernicious influence of free hydrochloric acid upon the structure of the teeth; but as we judge everything by contrast, and as the syrup of iron chloride is characterized by an absence of this one particular objectionable feature, I trust you will pardon me when I allude to the observations and conclusions of one or two other gentlemen who have experimented on the subject.

The results of the experiments of Dr. Smith,[1] of Edinburgh, Scotland, are interesting to both the physician and dentist. Dr. Smith experimented with eight of the compounds of iron, which at the time the experiments were made were most generally used as remedial agents. These were the saccharated carbonate of iron, twenty grains to an ounce of water; carbonate of iron, in the same proportions; syrup of the phosphate combined; the syrup of the iodide of iron and water, equal parts; the citrate of quinine and iron; the wine of iron; the sulphate; and the tincture of the chloride of iron. On examining the respective solutions after twenty-four hours, the teeth were found unaltered in those of the carbonate,

---

[1] *Edinburgh Medical Journal*, 1886, vol. ii. p. 631.

saccharated carbonate, phosphate, iodide, citrate, and the sulphate. In the solution of the wine of iron, the liquid itself was somewhat turbid; the teeth, however, appearing to be untouched. In that of the chloride of iron, a turbid sediment filled the bottom of the bottle and covered up the teeth from view; the fangs of the teeth were somewhat soft and flexible, and the enamel easily scraped down. The sediment under the microscope presented an amorphous appearance. In conclusion, Dr. Smith further says that, of his experiments, it would appear that certain preparations of iron, when directly applied, do exercise a powerful effect on the substance of the teeth; and the ratio of the effects obtained would seem to prove that, of all the preparations employed in these, that of the tincture of the chloride of iron acts more powerfully; the sulphate of iron next; and next to that again, although in comparison very immaterially, the wine of iron,—the other preparations of iron appearing to be inert.

At a somewhat later period, Dr. J. H. McQuillen, of Philadelphia, in speaking of the effect of this preparation of iron on the teeth, stated, in an article published in the *Dental Cosmos*,[1] that "a young lady, whose teeth, originally of excellent material, are now in the most dilapidated condition, informs me that she has taken the tincture of muriate of iron almost daily during the last year.

"In her case not only the affinity between the hydrochloric or muriatic acid and the lime in the teeth has been fully demonstrated, but, in addition, the impropriety of an indiscriminate use of that valuable remedy.

"Employed, as this agent sometimes is, for a week or weeks, with the direction to take fifteen drops every two hours in a little water, without any caution or corrective against its deleterious influence upon the teeth, it is no wonder that organs which are so important to health and appearance as the teeth should have their integrity seriously if not irremediably affected."

In connection with the direct action of the tincture upon the enamel, the experience of a physician of New York, Dr. E. M. Cameron, is worthy of more than a passing notice. Dr. Cameron, while visiting a sea-side resort a few summers ago, was called upon by a lady, suffering with intense pain, to have the right lower molar extracted. In the absence of a dentist, he succeeded, he said, in extracting the tooth. He was summoned again a few hours after

---

1 "The Deleterious Effects of the Muriate of Iron on the Teeth." Vol. viii. p. 345.

to see his patient, whom he found suffering with an excessive hemorrhage due to the extraction. Not having the usual styptic,—the tersulphate of iron,—he applied upon a pledget of cotton a small quantity of the tincture of the chloride. On calling upon the lady the following day, he was welcomed with the exclamation, "Doctor, you have stopped the hemorrhage, but you have destroyed two teeth!" On examining the adjacent teeth, he discovered that the enamel on the anterior and buccal surface of the second molar and on the posterior and buccal surface of the second bicuspid was softened to such an extent that it could be easily scraped off with the finger-nail. The case made such an impression on his mind that ever after, when he found it necessary to administer the tincture of the chloride of iron, he said he did so with a considerable degree of reluctance, and invariably cautioned his patients regarding its use, at the same time suggesting such precautions as he thought would best counteract its destructive influence.

One of the precautions generally suggested by the medical practitioner is to direct the patient to dilute with water. But chemical observation shows that water increases the destructive energy of the tincture of chloride of iron upon the structure of the teeth more than any other fluid. As an illustration, the effect of adding water to a simple solution of the chloride of iron is to give us basic salts of iron and the separation of free hydrochloric acid. If a freshly-extracted living tooth be immersed in the officinal tincture of the chloride,—say for a period of twenty-four hours,—we shall find that the enamel, the most susceptible portion of the tooth, is not injured in the slightest degree; but if the same tooth be immersed in the tincture diluted with water, in proportion of one to eight,—*i.e.* one part of the tincture and eight parts of water,—we shall find that the enamel is materially injured within a period of one minute. It is not, therefore, entirely a matter of the strength of the acid that is likely to injure the teeth, but a matter of fluidity or solubility. To illustrate still further: When a piece of zinc is immersed in strong sulphuric acid ($H_2SO_4$), it will be observed that the acid has no effect upon the structure of the zinc; but if a little water be added to the acid, we find that the zinc is immediately attacked by the acid. The zinc in the strong sulphuric acid is protected from destruction in the same manner that the enamel of the tooth, which is immersed in the strong tincture of chloride of iron, is protected,—viz., the surface is blocked up with the basic iron salts, insoluble in alcohol, which prevents chemical action. In case of the zinc, it is the sulphate of zinc resulting from the first action, which is insoluble in the concentrated acid, that forms a protecting coat over the surface of the zinc. The addition of water dissolves this protecting sulphate and renders further chemical action possible. In case of a tooth immersed in the pure tincture of the chloride of iron, a similar action takes place,—viz., the oxide of iron first formed protects the tooth from immediate chemical action, owing to its compact adherence to its surface. As additional testimony relating to the injurious action of the tincture of the chloride of iron upon the teeth, I might add that almost all authorities on therapeutics and materia medica have expressly laid down the fact that the acid and astringent preparations of iron act on the teeth with considerable energy.

It seems unnecessary, therefore, to cite any more cases, either for the purpose of showing the destructive tendency of the above-mentioned preparation of iron on the enamel of the teeth, or that physicians or dentists are ignorant of such a condition of affairs. It is now, I believe, generally admitted by almost every one who, I might add, has had an opportunity of observing the effects, that the tincture of the chloride of iron, although passing transiently through the mouth and over the surfaces of the teeth, nevertheless exerts a most powerful and pernicious action on their structure.

We will now consider the so-called indirect or secondary effect of acids on the fluids of the oral cavity.

It has been observed by one or two prominent gentlemen in the dental profession that, after taking the tincture of iron for a certain period of time, the saliva, ordinarily alkaline, became acid in reaction,[1] and the question has been raised whether such a condition of affairs might not seriously affect the integrity of the teeth. It is possible that the ingestion of a large quantity of acids, even in normal health, may decrease the alkalinity of the blood to such an extent that the glandular secretions of the mouth will be rendered more or less acid. I can only say that I have never been able, although I have taken a large quantity of the mineral acids, to produce such a change in the secretions of my own salivary glands.

We know that the urine, which is normally acid, is very susceptible of a change. But it must be borne in mind that the urine has a character altogether peculiar, and one which distinguishes it completely from every other animal fluid. All the others are either

---

[1] "Human Physiology," Dalton's fourth edition, p. 333; see Dr. Bogue's remarks, INTERNATIONAL DENTAL JOURNAL, August, 1891.

nutritive fluids, like the blood and milk, or are destined, like the secretions generally, to take some direct and essential part in the vital operations. Many of them, like the saliva and the gastric and pancreatic juices, are reabsorbed after they have done their work, and again enter into the current circulation. But the urine is merely a solution of excrementitious substances. There is a wide difference, accordingly, between the action of the kidneys and that of the true glandular organs, in which certain new and peculiar substances are produced by the action of the glandular tissue. The kidneys alone are purely excretory in their offices; and the urine is not intended to fulfil any function,—mechanical, chemical, or otherwise; but it is destined only to be eliminated and expelled. It is not surprising, therefore, that most medicinal and poisonous substances introduced into the circulation should be expelled from the body by the same channel. Those substances which tend to unite strongly with the animal matters, and to form with them insoluble compounds, such as the preparations of iron, lead, silver, arsenic, and mercury, are least liable to appear in the urine. They may occasionally be detected in this fluid when they have been given in large doses, but when administered in moderate quantity are not usually to be found there.

Most other substances, however, accidentally present in the circulation, pass off readily by the kidneys, either in their original form or after undergoing certain chemical modifications.

The salts of the organic acids, such as lactates, acetates, molates of sodium and potassium, when introduced into the circulation, are replaced by the carbonates of the same bases, and appear under that form in the urine. The urine accordingly becomes alkaline from the presence of carbonates whenever the above salts have been taken in large quantity, or after the ingestion of fruits and vegetables which contain them.

Bartholow,[1] in his treatise on Materia Medica and Therapeutics,

---

[1] "A Practical Treatise on Materia Medica and Therapeutics." Roberts Bartholow, M.A., M.D., LL.D., third edition, p. 87.

Professor Lehmann found that the urine exhibited an alkaline reaction a very few minutes after the administration of lactates and acetates. In one instance, by experimenting upon a person with congenital extroversion of the bladder,* in whom the orifices of the ureters were exposed, he found that the urine became alkaline in the course of seven minutes after the ingestion of half an ounce of acetate of potassium.

---

* "Physiological Chemistry," vol. ii. p. 133.

says that "the mineral acids, especially hydrochloric, have been proposed as remedies for ACUTE RHEUMATISM.

"The unquestionable utility of the tincture of the chloride of iron in rheumatism lends support to this practice. It is highly probable that the mineral acids check the formation of lactic acid in the blood." On another page the same author states,[1] "The mineral acids, when administered in medicinal doses, must on reaching the stomach act in accordance with their chemical position: they will combine with the bases and form salts; hydrochloric, and to a less degree phosphoric, aid digestion, acting as synergists to pepsin, and contribute to the formation of peptones. . . . It is true of all the mineral acids that their long-continued use diminishes the production of acid gastric juice, and in this way after a time they cause the very trouble for the relief of which they were originally administered. An acid solution on one side of an animal membrane and an alkaline solution on the other, is the condition most favorable to osmosis, hence the introduction of an acid into the stomach with sufficient quantity must impair the production of acid gastric juice. In practice this is found to be the case. The mineral acids are among the most diffusible substances known, and of these hydrochloric stands at the head; so much of these acids as do not enter into combination in the stomach diffuses quickly into the blood, and the salts which they form by combination with bases follow the laws of diffusion according to their class. The acids, especially the hydrochloric, and next nitric, diminish the alkalinity of the blood."

The researches of Garrod[2] are also interesting in connection with the subject. His researches appear to show that the pathology of certain forms of rheumatism, particularly gout, involved a peculiar morbid condition of the blood,—viz., an accumulation of uric acid, of which the blood in health contains a trace.

There is no authority, however, so far as I am informed, for assuming that the human saliva is ever rendered acid by the presence of acid in the blood; and because the urine is easily changed from an acid to an alkaline reaction, it in nowise follows that the saliva can be changed from an alkaline to an acid condition.

There appears to be but two ways to account for the statement

---

[1] "A Practical Treatise on Materia Medica and Therapeutics." Roberts Bartholow, M.A., M.D., LL.D., third edition, p. 83.

[2] In forty-seven cases of gout, an analysis of the blood-serum by the same experimenter showed a notable increase of this constituent.

recently made by Dr. Bogue,[1]—viz., that after taking the tincture of the chloride of iron he observed that "acid in his *saliva* was distinctly perceptible."

1. The presence of free acid in the blood.

2. A derangement of the secreting glands; the admixture of mucus; and the production of the *sympathetic saliva*[2] of the physiologists.

Regarding the first point, it may be said there is no chain of evidence from a physiological stand-point which affords any reason for believing that such a condition of affairs ever exists. Of course it is unsafe to say *a priori* that a certain thing cannot take place, especially when a careful and observing man like Dr. Bogue says that it does. But, as I recently stated in my lecture read before the Odontological Society of New York, I am unable to find any authority, and I know of no good reason for believing, that the secretions of the glands of the oral cavity can be rendered acid *by the ingestion of acids.*[3] It has been observed that in an animal where the alkalinity of the blood approaches even a neutral point the animal will die.

The presence of alkalies is essential to the oxidation of the organic constituents of this fluid,—in fact, as necessary to the life of the corpuscles as water is necessary to the life of a fish, or common air to the life of mankind.

Wright[4] found, on injecting various acids into the veins of

---

[1] See Dr. Bogue's remarks made before the Odontological Society, New York, and published in INTERNATIONAL DENTAL JOURNAL, August, 1891, p. 552.

[2] The reflex secretion of saliva by means of the *chorda tympani* nerve, etc.

[3] Wright states, it rarely happens that the salivary glands, when in a state of healthy activity, perform an excernent function to free the blood from any temporary impurity, unless the organs proper for this task shall fail in discharging it.

1. The reaction of the blood is alkaline owing to the presence of disodic phosphate, $NA_2$, $HPO_4$. (Maly.)

2. The alkalinity is less in persons suffering from anæmia, cachectic conditions, and chronic rheumatism. (Lépine.)

[4] See Piggott on saliva, *American Journal of Dental Science*, vol. iii. p. 342.

Piggott on morbid change of saliva, *American Journal of Dental Science*, vol. iii. p. 582.

Later experiment by Lehmann and Jacubowitsh have not supported all the results obtained by Wright.

The parotid saliva is obtained by placing a fine canula in Steno's duct (Eckhard); it has an alkaline reaction, but during the first few drops may healthy dogs, that in only one instance did the salivary glands excrete the injected acid. The subject of this observation was a strong bull-terrier dog, into the veins of which has been thrown three drachms of pyroligneous acid diluted with six ounces of water. At first the saliva became very frothy and alkaline, continued so for six hours, at the expiration of which time it was discharged very copiously, and found to contain acetic acid. Mitscherlich says that the reaction of the saliva is usually alkaline during a meal, and neutral or acid when fasting. This statement, which was first made by Mitscherlich,[1] has been confirmed by Marshall and Garrod. These latter observers are inclined to attribute this to the rapidity of the discharge during a meal, and the slowness with which the secretion is formed at other times. They found that ordinarily but two or three drops were discharged by one parotid gland in a quarter of an hour, and that was acid; but in half a minute after a morsel, reaction was neutral, and within the minute alkaline. This continued until twenty minutes after the meal, when it again became acid. *This is accounted for by the fact of the general acidity of the mucous surfaces of the mouth, which is sufficient to overpower the feeble alkalinity of the saliva when secreted in small quantity.*

Ziemssen,[2] in speaking of the acidity of the mouth in diabetes mellitus, says that the patient's *sense of taste* is commonly altered, being stale and clammy, and not infrequently, too, decidedly sweet, —the latter being due, probably, in many instances, to the presence of sugar, which, according to MacGregor, Nasse, Hetter, Lamperhoff,[3] is often found in the oral cavity. The transformation of the sugar into lactic acid is probably also the cause of the oral fluid in diabetics almost always showing an acid reaction. On another page the same authority, in speaking of the chemical alteration of the saliva during salivation, states that the saliva has been examined by many chemists,—Gmelin, Thomson, Simon, Ure,

---

be neutral or even acid on account of free $CO_2$ (Oehl); its specific gravity is 1003 to 1004. *Salivary calculi* are formed in the ducts of the salivary glands, owing to the deposition of lime-salts, and they contain only traces of the other salivary constituents; in the same way is formed the *tartar* of the teeth, which contains many threads of leptothrix, and the remains of low organisms which live in decomposing saliva in carious cavities between the teeth.

[1] Landois and Sterling's "Physiology," vol. i. p. 291.

[2] Ziemssen's "Encyclopædia of Medicine," vol. xvi. p. 911.

[3] "In Karth de Dyscrasia Saccharina Diss Brown," 1840; ibid., vol. vi. p. 853.

Bird, Garrod, Lehmann, and Wright,[1] have worked at this subject. At the commencement of the flow the specific gravity is usually very much increased, being as high as 1059, while it hardly reaches 1010 in normal saliva. This increase depends upon a greater admixture of mucus,—the sympathetic saliva of the physiologists,—some albumen, and an increase in the salts. In the course of the salivation, however, the specific gravity falls almost down to that of distilled water. Wright found it 1001.5 in one instance; with the subsidence of salivation the saliva usually acquires its normal characteristics again. Its reaction is either normal, slightly alkaline, or it is neutral or even slightly acid, due to the increase of the already souring mucus.

Landois and Sterling,[2] in speaking of the abnormal constituents of the saliva, state that decomposition products of epithelium salivary corpuscles, or the remains of food, may render it acid temporarily, as after long fasting and after much speaking (Hoppe-Seyler). Even outside the body the saliva containing much epithelium becomes acid before it putrefies (Gorup-Besanez). The reaction is acid in some cases of dyspepsia and in fever, owing to the stagnation and insufficient secretion; also that in diabetes mellitus, lactic acid, derived from a further decomposition of grape sugar, is found (Lehmann). It dissolves the lime in the teeth, giving rise to diabetic dental caries.

Foster[3] states that "the parotid saliva in man is clear and limpid, not viscid; the reaction of the first few drops secreted is often acid, the succeeding portions, at all events when the flow is copious, are alkaline,—that is to say, the natural secretion is alkaline; but this may be obscured by acid changes taking place in the fluid which has been retained in the duct, possibly by the formation of carbonic acid; . . . submaxillary saliva in man and in most animals differs from parotid saliva in being more alkaline and, from the presence of mucin, more viscid." Concerning the pathological aspects of saliva, Kingzett[4] states that the presence of lactic acid in saliva

---

[1] See *Lancet* for 1841 and 1842; Marshall and Garrod's experiments in same journal; G. Owens Rees on saliva, in the "Cyclopædia of Anatomy and Physiology;" Simon's "Chemistry of Man;" the article on Lehmann's "Physiological Chemistry," in the *British and Foreign Medico-Chirurgical Review* for October, 1851.

[2] Landois and Sterling's "Physiology," vol. i. p. 293.

[3] Foster's "Text-Book of Physiology," vol. ii. p. 362.

[4] Charles Thomas Kingzett, F.C.S., "Animal Chemistry." London, 1878, p. 53.

has been frequently asserted; but while there is great doubt about it, it is certain that during some diseases the saliva grows more or less acid; thus, according to Donne,[1] saliva is acid in inflammatory affections of the primæ viæ, in pleuritis, encephalitis, etc.

Wright assumes the acid state sometimes observed in saliva to be referable to, among other diseases, rachitis. But all these, and many similar observations, are not worth much credit, being too vague, and admitting of no reduction to definiteness. It is also said that lactic acid is to be found in saliva in cases of diabetes, but the statement has as often been contradicted.

Now, if Dr. Bogue, before experimenting, will wait until the first few stagnant drops of saliva have flowed off, he will undoubtedly find that the reaction of the saliva *per se* is alkaline.

The experiments of Dr. Henry L. Chase, of St. Louis, are worthy of consideration. Dr. Chase experimented and tested the reaction of the saliva of more than five hundred different animals. In a letter published in the *Dental Cosmos*[2] of 1868, correcting the report of the Committee on Chemistry, he says, "In the paper, I stated that I had found the mixed saliva of the mouth to be acid in nearly every person whom I had examined, being more than two hundred; . . . that I had found in every case, in a minute or two after a meal, the saliva to be either alkaline or neutral.

"That I consider a physiological condition of the mixed saliva to be either alkaline or neutral; that I found the saliva of the lower animals, such as horses, cows, etc., to be alkaline, provided they were living in a natural condition.

"That I found the saliva of cows, shut up where large numbers were herded together in stables, to be acid."

There is nothing expressed in Dr. Chase's monograph, however, which affords any good reason for assuming that the ingestion of acids, in medicinal doses, can in any way change the reaction of the saliva, although he makes the statement "that the saliva may be rendered *sour* by partaking inordinately of acids;" but he gives no proof. In justice to Dr. Chase, I have quoted his letter of explanation, for it materially modifies some of the statements made in his paper.

I do not question the sincerity or the results of his experiments, but I do question the method by which he obtained those results;

---

[1] "Histoire Physiol. et Pathol. de la Salive." Paris, 1836.

[2] *Dental Cosmos*, vol. x. p. 547, "Character and Uses of the Saliva," read before the American Dental Association, July, 1868.

for it must be observed that his experiments concern only the mixed saliva, and, of course, the first few stagnant drops, which, as I have already stated, are generally acid in reaction. If he will insert a fine canula into Steno's duct, and test the reaction of a small quantity of pure saliva, he will very probably admit that his paper, so far as it relates to the reaction of the saliva, is, to say the least, misleading.

Mention may be made of the bacteria theory of Miller[1] in connection with the disintegration of tooth-substance and the solvent action on their lime salts. Dr. Miller's experiments show:

1. "That fungi-bacteria constantly exists in the mouth, and especially in and around carious teeth, and as a resultant of their growth and increase a ferment-lactic-acid is produced.

2. "That these fungi can be isolated, their specific character determined, and their life-history studied.

3. "That artificial caries can be produced by placing teeth in a 'pure cultis' of these fungi, which cannot be distinguished microscopically from natural caries."

Dr. Magitot's[2] experiments are worthy of mention. He says, "It is perfectly established that dental caries results from the direct alteration of the organ by means of substances which originate in the saliva or are accidentally introduced,—an alteration usually preceded and favored by certain congenital or acquired predispositions of structure or anatomical conformation. Caries, regarded from this point of view," he says, "consists, then, precisely in a simple solution of the calcareous salts of the dental tissues by an acid element developed or brought in contact with them."[3] It would seem that this author places acids introduced into the oral cavity upon the same plane as those developed in contact with the teeth.

From carefully weighing all the effects that have been produced and all the doings that have been achieved in the matter, I am forced to conclude that the disintegration of tooth-structure in

---

[1] Dr. W. D. Miller, Berlin, "Fermentation in the Human Mouth: Its Relation to Caries of the Teeth."

Thanks are extended to Dr. A. L. Northrop, New York, for the privilege of access to his valuable library.

[2] See monograph of George S. Allan, D.D.S., New York, "Caries of the Teeth: Its Prevention and Treatment." Odontological Society, New York, April, 1886.

[3] See remarks of G. V. Black, M.D., D.D.S., "Dental Caries," INTERNATIONAL DENTAL JOURNAL, vol. i. p. 749.

conjunction with a chemical hypothesis, may be summed up as embracing two points, viz.:

1. The direct action of acids introduced from without. (The complete disintegration by a mineral acid applied at the very spot where the erosion had its beginning has already been alluded to in the cases mentioned by Dr. Cameron.)

2. The lodgement of food and the presence of bacteria in out-of-the-way points about the teeth, followed by decomposition, fermentation, and the evolution of acids thereof.

One word regarding erosion in connection with the acid-mucus theory and the affinity which exists between acids and the enamel and dentine. The idea that erosion is probably due to the solvent action of acid-mucus, secreted by the follicles situated in the *labial mucous membrane*, was first advanced by Professor James Truman. This theory has, I believe, been accepted by Drs. Black, Kirk, and a number of other authorities; and to account for the erosion which is sometimes found upon the lingual surfaces and the distal points of the teeth, it has been held that the follicles at the tip of the tongue also secrete an acid mucus.

All my experiments relating to the effect of acids upon lime-salts of the teeth demonstrate most emphatically that the enamel is more susceptible to the influence of all kinds of weak acids than the dentine, and that invariably the enamel will first be attacked and destroyed. Not infrequently, however, in cases of erosion, we find the *dentine* on the grinding surfaces of the teeth scooped out, so to speak, leaving a hollow cavity, which is surrounded by a thin edge of enamel, which, if we assume the acid mucus is the cause, should have first been destroyed. I touch upon this point now in view of Dr. Kirk's experiments and for his future consideration when studying the etiology of erosion and the cause of the acidity of the mucous follicles of the mouth.

Notwithstanding the objectionable features of the tincture of the chloride of iron,—viz., its peculiar and obnoxious taste, its liability in many cases to excite nausea and vomiting, and its extremely acid and astringent properties,—it is frequently employed by physicians, not only in the treatment of anæmia, but also in large doses in the treatment of erysipelas, neuralgia, diphtheria, epilepsy, acute rheumatism, and other disorders which might be mentioned. It acts also as a diuretic; even in ordinary anæmia many authorities prefer it to any other preparation, claiming that its effects are shown much more quickly, and that it seems to act more satisfactorily. Almost all agree that it possesses special virtues, either constitutional or local, which render it useful in the above-mentioned disorders. These peculiar properties (especially the diuretic effect) are ascribed by some to a peculiar ethereal compound, resulting from the mixture of the *liquor ferri chloridi* and the alcohol used in making the tincture, to which compound its odor is due.

As I stated in the beginning of this paper, the SYRUP OF IRON CHLORIDE is simply the tincture of the chloride of iron in a slightly modified form, a sufficient quantity of alkali having been added to counteract the free hydrochloric acid, and a small quantity of the syrup of gaultheria (which, it is claimed, in certain disorders, has a therapeutic value), to render it more palatable. In other words, the excess of acids over and above that necessary to form a perchloride of iron has been eliminated. The preparation is, therefore, acid in reaction; but the relative proportions of the acid and iron have been adjusted so delicately that, although sufficiently acid to prevent the precipitation of the basic salts of iron, it is not enough acid to injure the enamel of the teeth. And it is to be noted that, while the teeth are protected from injury, as soon as the preparation passes through the mouth into the stomach it meets with more hydrochloric acid,[1] and is likely returned again to its original condition, or nearly as it was in the original tincture of the chloride;[2] accordingly, it may be said that the therapeutic value of the syrup corresponds with that of the tincture. Moreover, it is claimed that the syrup as a restorative agent is easily assimilated and more likely to be tolerated by the mucous membrane of weak stomachs than the old form of the *acid chloride*, and is less harmful under prolonged use.

It is not claimed that all of the objectionable features of the tincture have been removed. For instance, the syrup possesses the same special and styptic qualities as that of the tincture, and it necessarily follows that there will be a correspondingly physiological effect produced on the mucous membrane of the bowels. It will be necessary, therefore, for the physician (as when using the tincture) to pay proper attention to this particular functional disorder.

It has been observed by Dr. Dana, of New York, when administering the syrup of iron chloride to children (when tonic remedies and supporting measures of treatment were indicated),

---

[1] It is supposed that hydrochloric acid is produced in the stomach by a reaction between the chloride of sodium and lactic acid.

[2] See Professor Elliott's remarks before New York Odontological Society, INTERNATIONAL DENTAL JOURNAL, August, 1891, p. 554.

that the tongue of the patient was less likely to be discolored than when the tincture was employed; and he has been enabled to more clearly define the presence of sordes. It is not claimed, however, that the *syrup* will not discolor the teeth. In reference to the latter, the same may be said of soft-boiled eggs and the various kinds of salad-dressings; but in all cases after the discoloration has been removed, the enamel and structure of the teeth are found to be intact.

The following cases have been reported to me by physicians who have employed the preparation in their practice:

Case I.—By Dr. Cyrus Edson, chief inspector, Board of Health, New York City.

Mrs. P., aged thirty-eight years, came to me suffering from a severe attack of anæmia and sciatica. She had been troubled with sciatic pains for four months previously, and although she had been under treatment by three other physicians, she had obtained no relief.

The etiology of the sciatica, as far as could be ascertained, was the history of severe grief, due to the loss of her son. I gave her an aperient (cascara sagrada) morning and evening, also ordered Weld's tincture of chloride of iron, to be taken three times a day, after meals, in four-drachm doses.

It is now one month since the last consultation. All pain has completely disappeared, and her blood is in excellent condition.

Case II.—Mr. C., age thirty-seven years; physical condition about the same as the patient in the previous case, though of much longer standing.

He has been troubled with sciatica, associated with anæmia, for about three years, but it is only within the last year that the pain has been continous.

I made use of subcutaneous injections of *aqua pura* along the course of the sciatic nerve; and also to improve the condition of the blood, Weld's tincture of iron.

The patient has been under my treatment for about a month, and complains now only of slight intermittent pain during the night. Physical appearance much improved.

Case III.—G. C., a child, eight years old, residence, Tremont, New York City, had a very bad attack of malarial fever followed by *anœmia*. I put him on Weld's tincture of iron, with the result of a decided amelioration of all the symptoms within two weeks.

In all the above cases the patients experienced no trouble in taking the preparation, and the teeth suffered no deleterious effect.

As far as my experience extends, I can say that Weld's preparation contains all the advantages, without any of the disadvantages, of the officinal "tinctura ferri chloridi."

Case IV.—By Dr. H. B. Bayles, sanitary inspector, Board of Health, Brooklyn, New York.

Mr. B. was taken with a chill on September 4, 1891, with slight elevation of temperature. The condition of moderate fever continued for several days, and developed, as was expected, into what was supposed to be a mild case of typhoid fever. In due time the eruptions occurred, and, aside from general debility, he did not complain of any very annoying symptoms; no profuse diarrhœa; in fact, being what might be termed comfortably sick.

This state of affairs continued till the sixteenth day of his illness, when he was attacked with epistaxis (not profuse); the next day there were extensive purpuric spots over the entire body, hemorrhages from the tongue and gums, and copious hemorrhages from the bladder. The temperature remained at 100.5° F., the pulse at 124,—the patient completely exsanguinated and in a critical condition. This state of affairs continued about one week. The treatment consisted of stimulants, heart tonics, and tinctura ferri chloridi. The iron was given in moderate doses and frequently; but the stomach, heretofore manageable, became rebellious, and rejected it. When the old reliable preparation of iron had to be abandoned, I considered my mainstay gone. Having the same day received a large bottle of the modified tincture of iron, prepared by Parke, Davis & Co., after the formula of Dr. G. W. Weld, of New York City, I immediately gave the same, commencing with one tablespoonful, and increasing to three tablespoonfuls every two hours. This preparation was tolerated by the stomach, gave rise to no nausea, and was taken continuously in varying doses for several weeks. I had the extreme satisfaction of seeing my patient restored to perfect health, and to learn that the old reliable tinctura ferri chloridi had been so modified that its greatest drawback had been removed, and that it was rendered an agreeable preparation.

Case V.—By Dr. A. H. Dana, New York City.

Louis L. had been suffering from typho-remittent fever for a period of five weeks. As usual in such cases, he was very much debilitated and in an anæmic condition. His stomach at the end of the fourth week was extremely irritable, and he was unable to retain anything in the way of food or medicine. Iron in some form was indicated. I used a number of preparations without success.

Having had my attention drawn to the syrup of iron chloride, I immediately prescribed it, giving him five tablespoonfuls each day. The effect was immediate: his nausea ceased, the digestive organs resumed their normal function, and he rapidly improved and regained his health.

I have since prescribed this preparation in three other cases with equally gratifying results.

FROM R. A. CALDWELL, M.D.

"24 WEST THIRTY-SECOND STREET, NEW YORK CITY.

"I have used the syrup of iron chloride with gratifying results. I believe it will prove a most valuable and permanent part of the armamentarium of our profession."

"15 WEST THIRTY-SEVENTH STREET,
"NEW YORK CITY, December 30, 1891.

"G. W. WELD, M.D.:

"DEAR SIR,—Permit me to say that I immersed a tooth, with perfect enamel, in the 'syrup of iron chloride,' for a period of two hours, and so far as I can see, the enamel has not been affected in the slightest degree.

(Signed) "GEORGE A. ALLAN, D.D.S."

FROM PARKER SYMS, M.D.

"55 WEST THIRTY-SIXTH STREET, NEW YORK CITY.

"The syrup of iron chloride possesses valuable points."

